# Sex differences in the structural rich-club connectivity in patients with Alzheimer’s disease

**DOI:** 10.3389/fnagi.2023.1209027

**Published:** 2023-09-13

**Authors:** Soo-Jong Kim, Youn Jung Bae, Yu Hyun Park, Hyemin Jang, Jun Pyo Kim, Sang Won Seo, Joon-Kyung Seong, Geon Ha Kim

**Affiliations:** ^1^Department of Neurology, Samsung Medical Center, Sungkyunkwan University School of Medicine, Seoul, Republic of Korea; ^2^Neuroscience Center, Samsung Medical Center, Seoul, Republic of Korea; ^3^Department of Intelligent Precision Healthcare Convergence, Sungkyunkwan University, Suwon, Republic of Korea; ^4^Department of Health Sciences and Technology, SAIHST, Sungkyunkwan University, Seoul, Republic of Korea; ^5^School of Biomedical Engineering, Korea University, Seoul, Republic of Korea; ^6^Alzheimer’s Disease Convergence Research Center, Samsung Medical Center, Seoul, Republic of Korea; ^7^Department of Digital Health, SAIHST, Sungkyunkwan University, Seoul, Republic of Korea; ^8^Department of Artificial Intelligence, Korea University, Seoul, Republic of Korea; ^9^Department of Neurology, Ewha Womans University College of Medicine, Seoul, Republic of Korea

**Keywords:** sex differences, structural brain network, network analysis, rich-club organization, thalamus

## Abstract

**Background and objectives:**

Alzheimer’s disease (AD) is more prevalent in women than in men; however, there is a discrepancy in research on sex differences in AD. The human brain is a large-scale network with hub regions forming a central core, the rich-club, which is vital to cognitive functions. However, it is unknown whether alterations in the rich-clubs in AD differ between men and women. We aimed to investigate sex differences in the rich-club organization in the brains of patients with AD.

**Methods:**

In total, 260 cognitively unimpaired individuals with negative amyloid positron emission tomography (PET) scans, 281 with prodromal AD (mild cognitive impairment due to AD) and 285 with AD dementia who confirmed with positive amyloid PET scans participated in the study. We obtained high-resolution T1-weighted and diffusion tensor images and performed network analysis.

**Results:**

We observed sex differences in the rich-club and feeder connections in patients with AD, suggesting lower structural connectivity strength in women than in men. We observed a significant group-by-sex interaction in the feeder connections, particularly in the thalamus. In addition, the connectivity strength of the thalamus in the feeder connections was significantly correlated with general cognitive function in only men with prodromal AD and women with AD dementia.

**Conclusion:**

Our findings provide important evidence for sex-specific alterations in the structural brain network related to AD.

## 1. Introduction

Alzheimer’s disease (AD), the most common type of dementia in older adults, is characterized by progressive cognitive decline (Ewers et al., 2011; Scheltens et al., 2016).

Epidemiological evidence suggests that the prevalence of AD dementia is higher in women than in men (Mazure and Swendsen, 2016). In addition to the higher prevalence of AD dementia in women, a longitudinal study demonstrated that cognitive progression in women with amnestic mild cognitive impairment (MCI) was twice as fast as that in men even after correcting for *APOE* genotypes (Lin et al., 2015). In addition, the annual conversion rate of MCI to AD dementia is higher in women than in men (Tifratene et al., 2015).

Cognitive abilities also demonstrate variations based on sex. Research indicates that men and women patients with MCI, even when experiencing similar levels of hippocampal atrophy and temporal lobe glucose metabolism, exhibit different verbal memory performance (Sundermann et al., 2016). Furthermore, there are notable sex differences in neuropsychiatric symptoms associated with AD dementia. Male AD dementia patients tend to display apathy, agitation, and socially inappropriate or abusive behavior, while female patients are more susceptible to depression, mood instability, and affective symptoms (Mazure and Swendsen, 2016; Ferretti et al., 2018).

However, the unpinning neuro-mechanisms responsible for the sex-related differences in AD remain unidentified. Only a few studies have reported on sex differences in the brain structures or cognitive decline in AD dementia (Ardekani et al., 2016; Koran et al., 2017). Women with positive AD biomarkers (Amyloid-β and tau) demonstrate faster rates of hippocampal atrophy and cognitive decline than do men (Koran et al., 2017). Conversely, some studies have demonstrated no distinct sex differences in post-mortem amyloid β or neurofibrillary tangle burden of patients with AD dementia (Barnes et al., 2005) or by the measurement of cerebrospinal fluid amyloid-β (Aβ42) and tau levels (Mattsson et al., 2017). The results exhibit some discrepancies. Therefore, more comprehensive research is required to investigate sex differences in the brain of patients with AD.

The human brain is organized into a large-scale network (Hagmann et al., 2007; Gong et al., 2009) characterized by an optimal balance between the integration and segregation of information (Latora and Marchiori, 2001). Hub regions, such as the superior frontal cortex, superior parietal cortex, and precuneus, play a pivotal role in the hierarchical organization of the brain (van den Heuvel and Sporns, 2011, 2013). These regions are highly interconnected with each other and form a central core, the rich-club (van den Heuvel and Sporns, 2011, 2013; van den Heuvel et al., 2013). Since rich-club connections link spatially distant regions, extensive long-distance white matter fibers to interconnect them are required. This incurs a high biological cost, it can be balanced out by having a substantial concentration of rich-club connections, thereby improving efficient communication (Van Den Heuvel et al., 2012).

In rich-club organization, the nodes are divided into rich-club and non-rich-club nodes, and then their connections are further classified into three categories including rich-club, feeder, and local connections (van den Heuvel and Sporns, 2011, 2013). The rich-club organization may play a pivotal role in cognitive function in the human brain (Griffa and Van Den Heuvel, 2018), and these rich-club brain regions are known to be involved in the integration of neural information (Kim and Min, 2020). Therefore, the investigation of rich-club organizations may provide structural network-based information relevant to the disease.

Disruptions in the structural brain network contribute to the pathophysiology of AD (Yan et al., 2018). Prior investigations into brain network connectivity in individuals with AD dementia have revealed disruptions in the overall level of structural connectivity, as well as significant lesions observed in hub regions located in the frontal, temporal, and parietal cortices (Dai et al., 2015; Fischer et al., 2015). In addition, a previous research related to the rich club organization in AD dementia have proposed that these structural network disruptions are particularly prevalent in regions that are more distantly connected within the brain (Daianu et al., 2015). Another study also found that disturbances in the rich-club organization have a dynamic and potent impact on the connectivity among peripheral regions in individuals with mild MCI (Yan et al., 2018). Moreover, these disrupted connectivity extend to the rich-club regions in the brains of patients with AD dementia (Yan et al., 2018). Notably, in comparison to rich-club connections, feeder and local connections are shown to exhibit earlier and more severe impairments in patients with AD dementia (Daianu et al., 2015).

However, whether alterations in the rich-club organization in the AD spectrum differ between sexes remains relatively unknown. Although previous studies have investigated the sex differences of rich-club organizations in healthy young participants (Wang et al., 2019), no studies have yet explored this aspect within AD patients. Furthermore, even though several prior investigations have examined the rich-club organization in AD dementia (Dai et al., 2015; Yan et al., 2018), these studies have not taken into account the topological attributes of the rich-club organization. Because the topological features of the rich-club organization provides valuable insights into the fundamental principles governing brain connectivity related to cognitive function, investigating topological characteristics of rich-club organization is also important to understand the sex-difference in the rich-club organization in patients with AD.

Therefore, we aimed to investigate sex differences in the rich-club organization of the brain in AD and to explore the association between sex differences in the relationship between rich-club organization and cognition.

## 2. Materials and methods

### 2.1. Participants

The study cohort included 826 participants; 260 of them were cognitively unimpaired (CU), 281 with prodromal AD, and 285 with AD dementia.

All participants underwent a comprehensive assessment including, neuropsychological evaluation, amyloid positron emission tomography (PET) using either flutemetamol (FMM) or florbetaben (FBB), and brain T1-weighted magnetic resonance imaging (MRI). Furthermore, each participant underwent clinical interviews encompassing clinical dementia ratings (CDR) (Morris, 1997; Choi et al., 2001), neurological and neuropsychological examinations, as well as laboratory tests. Neuropsychological evaluation utilized the Seoul Neuropsychological Screening Battery 2nd edition (SNSB-II) which encompasses five cognitive domains: attention, memory, visuospatial, language and frontal executive function (Ryu and Yang, 2023). Additionally, laboratory assessments included a complete blood count, blood chemistry, thyroid function tests, syphilis serology, and vitamin B12/folate levels. To confirm the absence of structural lesions, including cerebral infarctions, brain tumors, vascular malformations, and hippocampal sclerosis, brain MRI was conducted.

Individuals with CU status met the following criteria: (1) absence of medical history likely to impact cognitive function, as per Christensen’s health screening criteria (Christensen et al., 1991); and (2) absence of objective cognitive impairment across various domains, determined by a comprehensive neuropsychological test battery (scoring above −1.0 standard deviations (SD) of age-matched and education-matched norms in memory, and above −1.5 SD in other cognitive domains), as assessed using SNSB-II (Ryu and Yang, 2023). For the purposes of this study, only CU individuals with negative amyloid PET scans were included. Participants diagnosed with amnestic MCI needed to satisfy the subsequent criteria (Petersen et al., 2014): (1) self-reported cognitive complaints by participants or caregivers; (2) objective cognitive impairment in at least one memory test (scoring below −1.0 SD of age-matched and education-matched norms in memory); (3) absence of significant impairment in activities of daily living; and (4) no diagnosis of dementia. In this study, only individuals with MCI and positive amyloid PET scans were chosen (Albert et al., 2011), referred to as prodromal AD henceforth. Participants with AD dementia were categorized as having probable AD dementia with positive amyloid PET scan, based on the National Institute on Aging-Alzheimer’s Association Research Criteria for AD (McKhann et al., 2011).

Written informed consent was obtained from all participants. The Institutional Review Board of Samsung Medical Center approved the study protocol, and all methods were performed according to the approved guidelines.

### 2.2. Imaging data acquisition: MRI/PET

#### 2.2.1. MRI data acquisition

Standardized 3-D T1 turbo field echo images were acquired from all participants at the Samsung Medical Center using an identical 3.0T MRI scanner (Philips Achieva; Philips Healthcare, Andover, MA, USA). The 3-D T1 parameters were as follows: sagittal slice thickness, 1.0 mm over contiguous slices with 50% overlap; no gap; repetition time (TR), 9.9 ms; echo time (TE), 4.6 ms; flip angle, 8°; and matrix size, 240 × 240 pixels reconstructed to 480 × 480 over a field of view (FOV) of 240 mm. In the whole-brain diffusion-weighted MRI examinations, we collected sets of axial diffusion-weighted single-shot echo-planar images using the following parameters: 128 × 128 acquisition matrix, 1.72 × 1.72 × 2 mm^3^ voxels; reconstructed to 1.72 × 1.72 × 2 mm^3^; 70 axial slices; 220 × 220 mm^2^ FOV; TE 60 ms, TR 7,383 ms; flip angle 90°; slice gap 0 mm; and b-factor of 600 s/mm^2^. Using the baseline image without diffusion weighting (reference volume), diffusion-weighted images were acquired from 45 directions. All axial sections were acquired parallel to the anterior commissure-posterior commissure line.

#### 2.2.2. Amyloid β PET data acquisition

The participants underwent FMM and FBB PET at the Samsung Medical Center using a Discovery STe PET/CT scanner (GE Medical Systems, Milwaukee, WI, USA) in 3-D scanning mode that examined 3.3-mm-thick 47 slices spanning the entire brain. According to the protocols for the ligands proposed by the manufacturers, a 20-min emission PET scan in dynamic mode (consisting of 4 × 5 min frames) was performed 90 min after injecting a mean dose of 185 MBq FMM or 311.5 MBq FBB. We reconstructed 3-D PET images in a 128 × 128 × 47 matrix with a voxel size of 2 × 2 mm × 3.27 mm using the ordered-subsets expectation maximization algorithm (FMM iterations = 4 and subset = 20; FBB iterations = 4 and subset = 20). The cut-off values of amyloid β positivity were 1.03 for FMM and 1.1 for FBB, which were defined by an iterative outlier method in both FMM and FBB PET (Kim et al., 2021).

### 2.3. Network construction

To construct the white matter networks, we followed the DTI process described by Lee et al. The processing details have been described previously (Lee et al., 2018). Briefly, the T1-weighted image was linearly registered to the reference volume of the diffusion image using FMRIB’s Linear Image Registration Tool and nonlinearly registered to ICBM152 in the Montreal Neurological Institute space, where the automated anatomical labeling (AAL) template was defined using fast nonlinear image registration in the FSL program. The AAL atlas was mapped to an individual’s diffusion space using the T1 transformation parameters. We corrected all diffusion-weighted images for eddy current distortion and head motion. A diffusion toolkit was used to perform the deterministic tractography. After fiber tracking, the networks were constructed using AAL templates for a 90 × 90 connectivity matrix.

### 2.4. Assessment of global network metrics

Network analysis was performed using the Brain Connectivity Toolbox.^1^ We computed the connectivity strength, global efficiency (E_glob_), local efficiency (E_loc_), and clustering coefficient in each matrix to assess the graph metrics of the global topological organization of the whole-brain structural connectivity network. The connectivity strength is defined as the sum of all streamlines connecting nodes (Rubinov and Sporns, 2010). Global efficiency is a measure of the network integration and defined as the average inverse shortest path length between all pairs of nodes (Rubinov and Sporns, 2010). The mean clustering coefficient of a network is calculated as the average of the clustering coefficients across all nodes (Rubinov and Sporns, 2010).

### 2.5. Assessment of rich-club organization

Rich-club nodes were selected according to previously published guidelines (van den Heuvel and Sporns, 2011; Yoon et al., 2016). The selected rich-club nodes included the superior frontal cortex (SFC), superior parietal cortex (SPC), precuneus, hippocampus, putamen, and thalamus. Briefly, we computed the normalized rich-club coefficient to demonstrate the existence of rich-club organization in a network. This is because random networks demonstrate increasing network connections because nodes with a higher degree have a higher probability of being interconnected by chance. The rich-club coefficient, Φ (κ), is typically normalized relative to a set of comparable random networks of equal size and similar connectivity distribution (van den Heuvel and Sporns, 2011). Thus, for each network, *m* = 1,000 random networks were computed by shuffling the links, preserving the weights and degree sequence, and, thus, all node degrees, including the hubs in the network. Similar to that in other previous studies (van den Heuvel et al., 2013), a normalized rich-club coefficient Φ norm (κ) >1 over a range of κ indicated a rich-club organization in a network. A rich-club organization was identified by performing a two-tailed t-test with permutation testing (10,000 permutations) of the area under the curve (normalized weighted rich-club coefficient against degree), compared with a random network (Supplementary Figure S1).

Rich-club nodes are likely to be highly connected with each other, compared with connections expected by random chance (van den Heuvel and Sporns, 2011). All connections between the nodes in each structural matrix were categorized into one of the following groups: “rich-club connections,” which are defined as the number of connections associating rich-club nodes, “feeder connections,” which are defined as the number of connections associating rich-club to non-rich-club nodes, and “local connections,” which are defined as the number of connections associating non-rich-club nodes (van den Heuvel and Sporns, 2011). To examine the region-specific alterations in the rich-club connections according to the cognitive status, we computed the connectivity strength of each rich-club node related to other nodes. Rich-club connections in the SFC, SPC, precuneus, putamen, and thalamus were calculated for each hemisphere and averaged into one value for each node.

For visualization, we converted the raw values of the connectivity strength of the rich-club organizations to a standardized Z-score using the mean scores and standard deviations (SD) of the CU group.

### 2.6. Statistical analyses

For baseline demographic data, we performed independent *t*-tests and chi-square tests for the continuous and dichotomous variables, respectively. We assessed the group differences in cognitive function as well as network characteristics using an analysis of covariance with the age and years of education as the covariates.

For sex effects on the global network metrics, including the connectivity strength, global efficiency, and the clustering coefficient of the whole-brain structural connectivity network, were compared between men and women using a general linear model (GLM) after adjusting for their age and years of education. We also used the GLM to examine the differences in the rich-club, feeder, and local connections between men and women, using the same covariates. Partial eta squared (*η_p_^2^*) was used to estimate the effect size. In addition, we examined the interaction effects between sex and cognitive groups on the rich-club organization. The Pearson’s correlation analysis was used to examine the relationship between the rich-club organization and cognitive performance including the scores of Korean-Mini-Mental Status Examination (K-MMSE) and the sum of boxes of CDR. For the correlation analyses, we excluded outliers beyond the 5th to 95th percentile range of the K-MMSE scores or the sum of boxes of CDR.

We employed a permutation-based multiple testing method to investigate the effects of group and sex on the rich-club organization (Nichols and Holmes, 2002; Hurtz et al., 2014). In this approach, we randomly permutated the sex or group status of subjects while maintaining the age and levels of education values. Data were assessed against an empirical null distribution by running 10,000 synthesized permutations with a threshold of *p* < 0.05. A permutation-adjusted *p* value was computed based on the proportion of permutations with *p* values under the null distribution that was greater than the observed values from the actual data set (Westfall et al., 1993).

All analyses were performed using STATA 17.0 (StataCorpx, College Station, TX, USA).

## 3. Results

### 3.1. Basic information

#### 3.1.1. Clinical characteristics

We observed no significant sex differences in the mean age, K-MMSE scores, CDR, or CDR sum of boxes among the whole participants (Table 1), whereas men exhibited higher educational levels than those of women in all groups.

**Table 1:** Demographic characteristics.

	Women	Men	
	(*n* = 504)	(*n* = 322)	*p*-value
Age	69.92 ± 8.03	70.78 ± 8.54	0.147
Education	10.34 ± 4.73	13.69 ± 4.01	<0.001*
DM (n, %)	71 (14.09)	63 (19.57)	0.033*
HTN (n, %)	193 (38.29)	144 (44.72)	0.054
HL (n, %)	176 (34.92)	101 (31.36)	0.313
APOE4 carrier (n, %)	243 (44.64)	159 (46.27)	0.088
K-MMSE	23.72 ± 5.87	24.31 ± 5.11	0.143
CDR	0.68 ± 0.50	0.67 ± 0.43	0.713
Group (n, %)			0.503
CU	166 (32.94)	94 (29.19)	
Prodromal AD	166 (32.94)	115 (35.71)	
AD dementia	172 (34.13)	113 (35.09)	

APOE4, Apolipoprotein E4; CDR, Clinical dementia rating; K-MMSE, Korean-Mini-Mental Status Examination; CU, cognitively unimpaired; DM, diabetes mellitus; HL, hyperlipidemia; and HTN, hypertension. *p-value < 0.05.

According to each cognitive group (Table 2), there were no significant differences in the clinical severity and amyloid deposition in all groups. In addition, we observed no significant sex differences in the global network topologies, including the connectivity strength, clustering coefficient, and global efficiency, in the CU, prodromal AD, and AD dementia groups.

**Table 2:** Clinical characteristics according to the cognitive group.

	CU			Prodromal AD			AD Dementia		
	Women	Men	*p*-value for sex	Women	Men	*p*-value for sex	Women	Men	*p*-value for sex
	(*n* = 166)	(*n* = 94)		(*n* = 166)	(*n* = 115)		(*n* = 172)	(*n* = 113)	
Age	69.10 ± 6.85	69.88 ± 7.88	0.411	71.28 ± 7.88	72.27 ± 7.54	0.292	69.40 ± 9.05	69.99 ± 9.81	0.599
Education	10.83 ± 4.75	13.11 ± 4.43	0.0002*	10.48 ± 4.83	13.60 ± 3.77	<0.001*	9.74 ± 4.59	14.28 ± 3.83	<0.001*
DM (n, %)	29 (17.46)	20 (21.27)	0.389	20 (12.05)	26 (22.61)	0.02*	22 (12.79)	17 (15.04)	0.560
Hypertension (n, %)	65 (39.16)	43 (45.74)	0.213	63 (37.95)	53 (46.08)	0.193	65 (38.01)	48 (42.47)	0.381
Hyperlipidemia (n, %)	66 (39.76)	31 (32.98)	0.336	60 (36.14)	32 (27.83)	0.129	50 (29.07)	38 (33.63)	0.376
*APOE4* carrier (n, %)	19 (11.44)	20 (21.27)	0.008*	101 (60.84)	72 (62.61)	0.206	105 (61.05)	57 (50.44)	0.200
Amyloid PET (SUVR)	0.92 ± 0.05	0.92 ± 0.05	0.613	1.40 ± 0.19	1.45 ± 0.22	0.029	1.49 ± 0.20	1.49 ± 0.16	0.984
Cognitive function and severity									
K-MMSE	28.27 ± 1.85	28.51 ± 1.35	0.271	24.6 ± 3.39	25.37 ± 3.15	0.068	17.92 ± 5.91	19.59 ± 5.09	0.019*
CDR	0.40 ± 0.20	0.41 ± 0.20	0.905	0.53 ± 0.16	0.53 ± 0.17	0.851	1.11 ± 0.63	1.03 ± 0.51	0.265
CDR, sum of boxes	0.63 ± 0.47	0.63 ± 0.48	0.997	2.15 ± 1.68	1.80 ± 1.35	0.066	6.54 ± 3.44	6.23 ± 2.99	0.453
Global brain network properties									
Connectivity Strength	4.85 ± 0.68	4.96 ± 0.73	0.238	5.13 ± 0.55	5.15 ± 0.58	0.802	4.64 ± 0.61	4.62 ± 0.56	0.804
Clustering Coefficient	0.17 ± 0.02	0.18 ± 0.02	0.300	0.18 ± 0.02	0.19 ± 0.01	0.083	0.17 ± 0.02	0.17 ± 0.02	0.132
Global Efficiency	0.19 ± 0.02	0.20 ± 0.03	0.323	0.20 ± 0.02	0.20 ± 0.02	0.958	0.19 ± 0.02	0.19 ± 0.02	0.958

APOE4, Apolipoprotein E4; DM, diabetes mellitus; CDR, Clinical dementia rating; SUVR, Standardized Uptake Value Ratio; PET, positron emission tomography; and K-MMSE, Korean-Mini-Mental Status Examination. *P < 0.05.

### 3.2. Rich-club organization

#### 3.2.1. Sex and group effects in the rich-club organization

**Figure 1:**
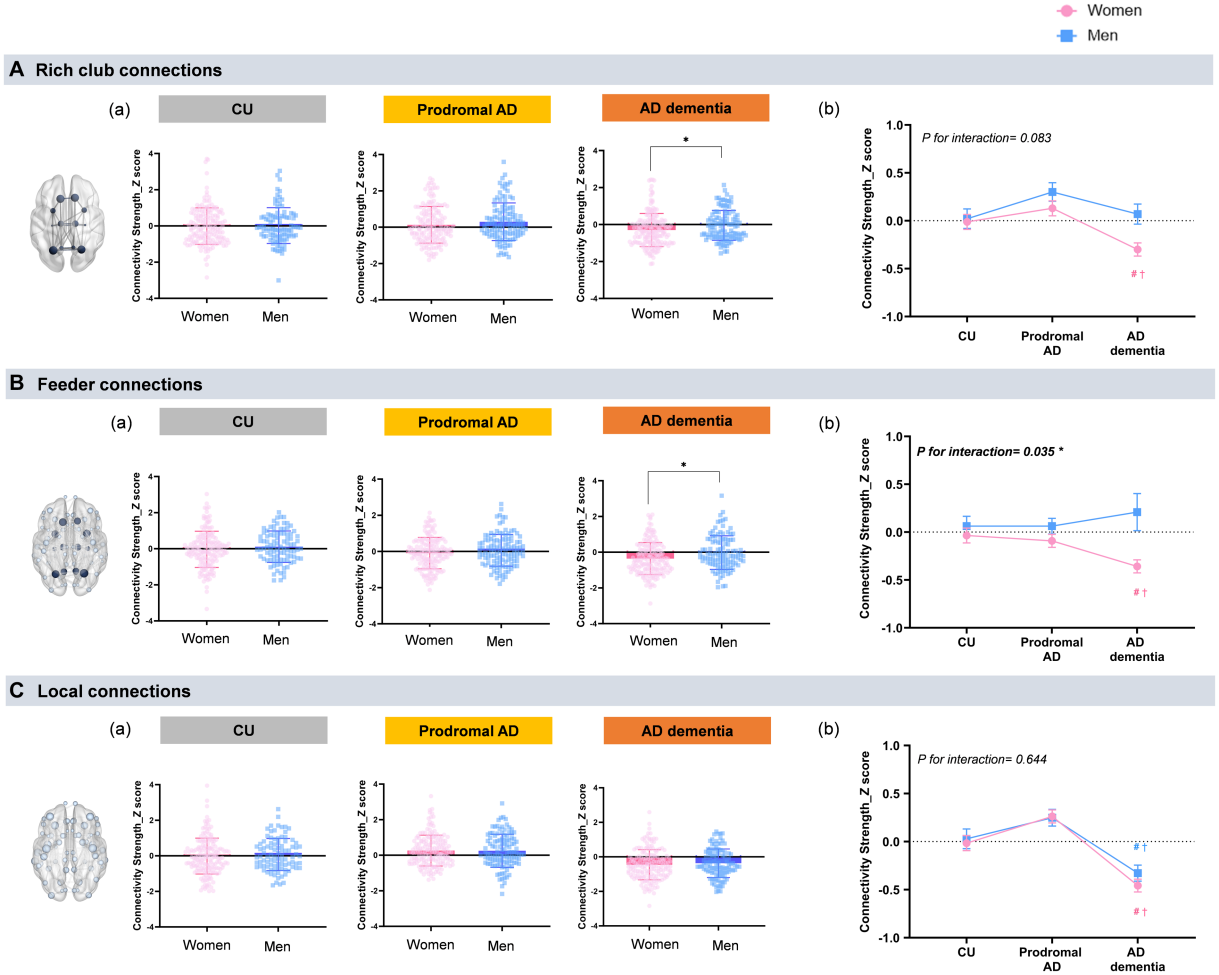
Sex and group differences in the rich-club organization. **(a)** Shows sex differences of rich-club connections in each group (the mean values with SD). A general linear model was used to examine the differences in the rich-club, feeder, and local connections between men and women with age and education of years as covariates. The rich-club nodes included the superior frontal cortex (SFC), superior parietal cortex (SPC), precuneus, hippocampus, putamen, and thalamus. *P < *0.05 for sex-differences*. **(A)** Rich club connections: Only the AD dementia group demonstrates sex differences in the rich-club connections, which indicate women show a lower connectivity strength of the rich-club connections than men (−0.29 ± 0.89 vs. −0.0 ± 0.76, *η_p_^2^* = 0.03, permutation-adjusted *p* = 0.034). **(B)** Feeder connections: Only women with AD dementia show lower connectivity strength of the feeder connections (−0.36 ± 0.89 vs. −0.02 ± 0.94, *η_p_^2^* = 0.03, permutation-adjusted *p* = 0.01) than men. There were no sex-differences in the other groups. **(C)** Local connections: No sex-related differences are observed in the local connections in the all cognitive groups. **(b)** Shows the mean values with SE of connectivity strength in the rich-club organizations that were obtained by GLM after adjusting age and years education. #*p* < 0.05: CU vs. AD; †*p* < 0.05 for prodromal AD vs. AD dementia. **(A)** Rich club connections: Women with AD dementia demonstrate significantly decreased connectivity strength in the rich-club connections (*η_p_^2^* = 0.04, permutation-adjusted *p* < 0.001) compared with CU or prodromal AD, whereas there were no group differences of rich-club connections in men (*η_p_^2^* = 0.02, permutation-adjusted *p* = 0.160). There were no significant group-by-sex interaction in the rich-club connections (*P for interaction = 0.083*). **(B)** Feeder connections: Women with AD dementia also show significantly decreased connectivity strength in the feeder connections (*η_p_^2^* = 0.03, permutation-adjusted *p* < 0.001) compared to those in CU or in prodromal AD, while there were no group differences of feeder connections in men (*η_p_^2^* = 0.001, permutation-adjusted *p* = 0.360). A significant group-by-sex interaction is observed in the feeder connections (*P for interaction = 0.035*). **(C)** Local connections: Both women and men with AD dementia show lower connectivity strength in the local connections (*η_p_^2^* = 0.106, permutation-adjusted *p* < 0.001) compared with CU or prodromal AD. There were no significant group-by-sex interaction in the rich-club connections (*P for interaction = 0.644*).

Figure 1A shows sex differences of rich-club connections in each group (the mean values with SD). In a whole participants, women demonstrated lower connectivity strength of the rich-club connections (mean ± SD, −0.09 ± 0.76 vs. 0.04 ± 0.74, *η_p_^2^* = 0.01, permutation-adjusted *p* = 0.009) and feeder connections (−0.15 ± 0.74 vs. −0.01 ± 0.73, *η_p_^2^* = 0.02, permutation-adjusted *p* = 0.001) than did men. Particularly in the AD dementia group, women demonstrated a lower connectivity strength of the rich-club connections (−0.29 ± 0.89 vs. −0.0 ± 0.76, *η_p_^2^* = 0.03, permutation-adjusted *p* = 0.034) and feeder connections (−0.36 ± 0.89 vs. −0.02 ± 0.94, *η_p_^2^* = 0.03, permutation-adjusted *p* = 0.01) than did men.

Figure 1B shows the mean values with SE of connectivity strength in the rich-club organizations after adjusting age and years education. For the group effects, women in the AD dementia group demonstrated significantly lower rich-club (*η_p_^2^* = 0.04, permutation-adjusted *p* < 0.001), feeder connections (*η_p_^2^* = 0.03, permutation-adjusted *p* < 0.001), and local connections (*η_p_^2^* = 0.11, permutation-adjusted *p* < 0.001) than those in the CU or prodromal AD group. However, men in the AD dementia group demonstrated only significant differences in the connectivity strength in the local connections, compared with those in the CU or prodromal AD group (*η_p_^2^* = 0.07, permutation-adjusted *p* < 0.001). Conversely, there were no significant differences in the connectivity strength between the rich-club (*η_p_^2^* = 0.02, permutation-adjusted *p* = 0.160) and feeder connections (*η_p_^2^* = 0.001, permutation-adjusted *p* = 0.360), according to the cognitive status in men. We observed a significant group-by-sex interaction only in the feeder connections (*P for interaction* = 0.035).

#### 3.2.2. Sex and group effects in the region-specific alterations of rich-club connections

**Figure 2:**
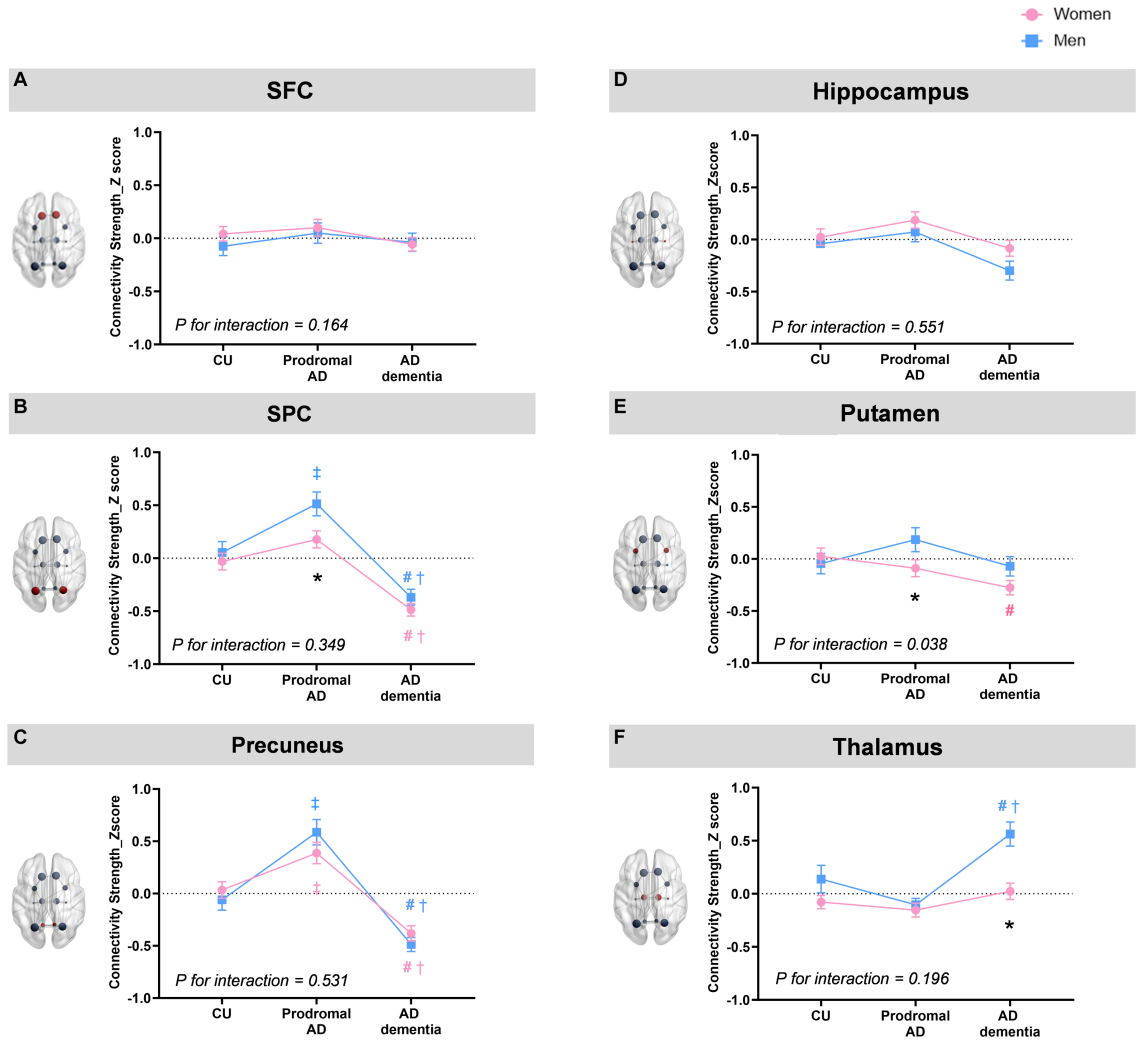
Sex and group effects in the region-specific alterations of rich-club connections. When we investigated the region-specific alterations in the rich-club connections between men and women, men show significantly greater connectivity strength of the SPC with other rich-club nodes (mean ± SE,0.43 ± 0.09 vs. 0.15 ± 0.07, *η_p_^2^* = 0.01, permutation-adjusted *p* = 0.021) as well as that of the putamen with other rich-club nodes (0.19 ± 0.12 vs. −0.09 ± 0.08, *η_p_^2^* = 0.01, permutation-adjusted *p* = 0.039) in the prodromal AD **(B,E)**. In AD dementia group, the connectivity strength between the thalamus and other rich-club nodes was significantly higher in the men compared to that women (0.56 ± 0.11 vs. 0.02 ± 0.08, *η_p_^2^* = 0.02, permutation-adjusted *p* = 0.001) **(F)**. For the group effects, both men and women showed higher connectivity strength of the precuneus in the prodromal AD group compared to that in CU (*η_p_^2^* = 0.08, permutation-adjusted *p* <0.001 for women; *η_p_^2^* = 0.16, permutation-adjusted *p* = <0.001 for men) but lower connectivity strength in AD dementia (*η_p_^2^* = 0.08, permutation-adjusted *p* <0.001 for women; *η_p_^2^* = 0.16, permutation-adjusted *p* <0.001 for men) compared to CU or prodromal AD groups **(C)**. Furthermore, both women and men demonstrate decreased connectivity strength of the SPC (*η_p_^2^* = 0.08, permutation-adjusted P <0.001 for women; *η_p_^2^* = 0.12, permutation-adjusted *P* <0.001 for men) in AD dementia group compared to CU or prodromal AD **(B)**. Especially among women, the connectivity strength of the putamen in AD dementia was decreased compared to CU (*η_p_^2^* = 0.02, permutation-adjusted *p* = 0.02) **(E)**, whereas among men, the connectivity strength of the thalamus in AD dementia was increased among men compared to CU or prodromal AD groups (*η_p_^2^* = 0.01, permutation-adjusted *P* = 0.02) **(F)**. There was a significant group by sex interaction on the connectivity strength of putamen with other rich-club node (*η_p_^2^* = 0.013, *P* = 0.026). * *Adjusted p* < 0.05 for sex-difference; # *Adjusted p* < 0.05: CU vs. AD dementia; ‡ *Adjusted p* < 0.05 for CU vs. Prodromal AD; † *Adjusted p* < 0.05 for prodromal AD vs. AD dementia.

Figure 2 indicates sex and group effects in the region-specific alterations of rich-club connections (mean ± SE). When we investigated the region-specific alterations in the rich-club connections between men and women, men showed significantly greater connectivity strength of the SPC with other rich-club nodes (0.43 ± 0.09 vs. 0.15 ± 0.07, *η_p_^2^* = 0.01, permutation-adjusted *p* = 0.021) as well as that of the putamen with other rich-club nodes (0.19 ± 0.12 vs. −0.09 ± 0.08, *η_p_^2^* = 0.01, permutation-adjusted *p* = 0.039) in the prodromal AD (Figures 2B,E). In AD dementia group, the connectivity strength between the thalamus and other rich-club nodes was significantly higher in the men compared to that of women (0.56 ± 0.11 vs. 0.02 ± 0.08, *η_p_^2^* = 0.02, permutation-adjusted *p* = 0.001) (Figure 2F).

For the group effects, both men and women showed higher connectivity strength of the precuneus in the prodromal AD group compared to that in CU (*η_p_^2^* = 0.08, permutation-adjusted *p* < 0.001 for women; *η_p_^2^* = 0.16, permutation-adjusted *p* = <0.001 for men) but lower connectivity strength of the precuneus in AD dementia (*η_p_^2^* = 0.08, permutation-adjusted *p* < 0.001 for women; *η_p_^2^* = 0.16, permutation-adjusted *p* < 0.001 for men) compared to CU or prodromal AD groups (Figure 2C). Furthermore, both women and men demonstrate decreased connectivity strength of the SPC (*η_p_^2^* = 0.08, permutation-adjusted *p* < 0.001 for women; *η_p_^2^* = 0.12, permutation-adjusted *p* < 0.001 for men) in AD dementia group compared to CU or prodromal AD (Figure 2B). Especially among women, the connectivity strength of the putamen in AD dementia was decreased compared to CU (*η_p_^2^* = 0.02, permutation-adjusted *p* = 0.02) (Figure 2E), whereas among men, the connectivity strength of the thalamus in AD dementia was increased among men compared to CU or prodromal AD groups (*η_p_^2^* = 0.01, permutation-adjusted *p* = 0.02) (Figure 2F). There was a significant group by sex interaction on the connectivity strength of putamen with other rich-club node (*η_p_^2^* = 0.013, *p* = 0.026).

#### 3.2.3. Sex and group effects in the region-specific alterations of feeder connections

**Figure 3:**
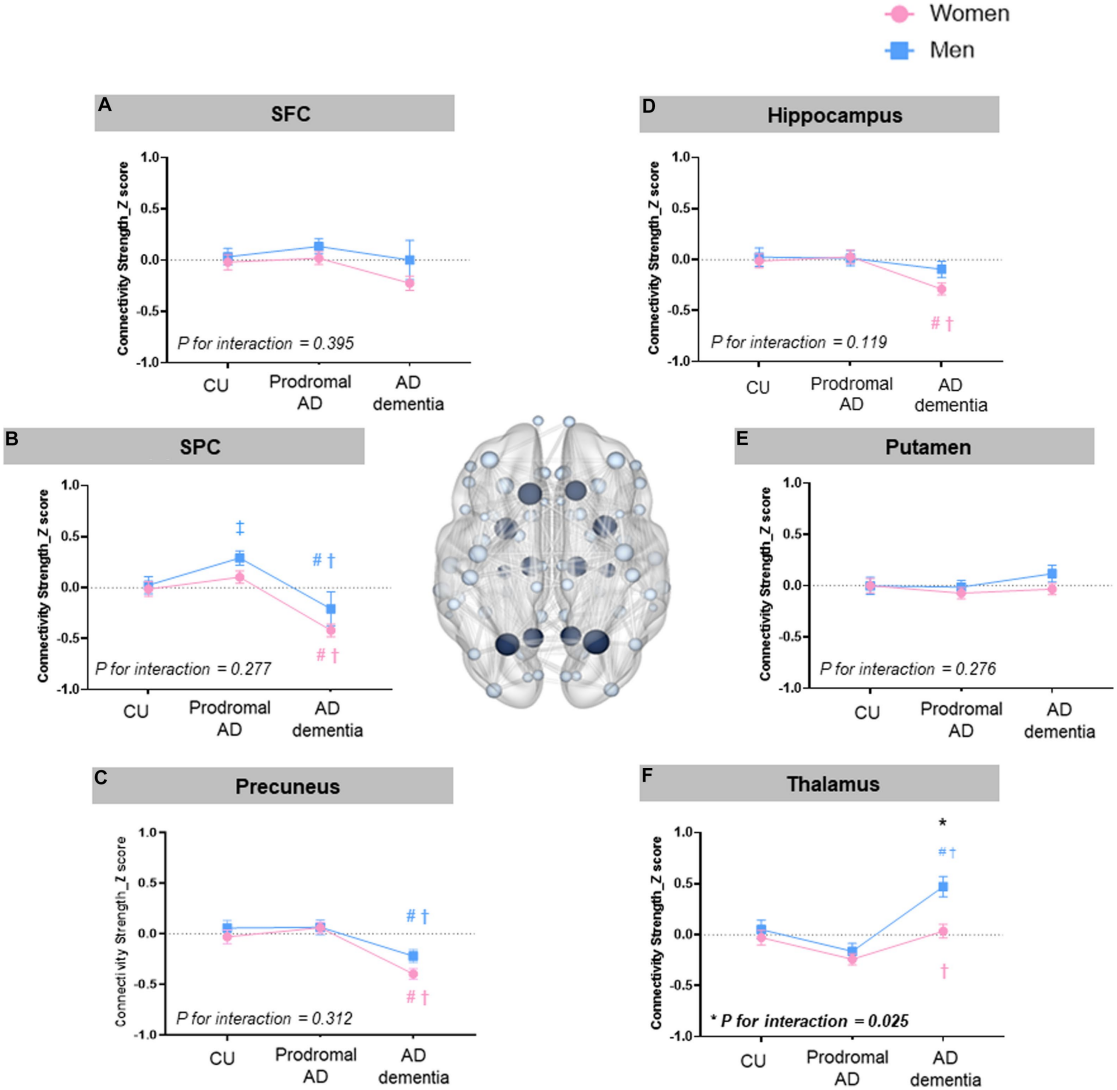
Sex and group effects in the region-specific alterations of feeder connections. There were significant sex-differences in the connectivity strength of the thalamus among the AD dementia group (mean ± SE, 0.03 ±0.07 for women vs. 0.44 ± 0.9 for men, *η_p_^2^* = 0.02, permutation-adjusted *p* = 0.026). For the group effects, both men and women with AD dementia showed decreased connectivity strength of SPC (*η_p_^2^* = 0.07, permutation-adjusted *p* <0.001 for women; *η_p_^2^* = 0.13, permutation-adjusted *p* <0.001 for men) and that of precuneus (*η_p_^2^* = 0.07, permutation-adjusted P <0.001 for women; *η_p_^2^* = 0.03, permutation-adjusted *p* =0.01 for men) compared to CU and prodromal AD (Figures 2B,C). Especially among women, the connectivity strength of hippocampus in the feeder connection was decreased in AD dementia (*η_p_^2^* = 0.03, permutation-adjusted *p* <0.001) compared to CU or prodromal AD. Among men, the connectivity strength of SPC in the feeder connections was increased in the prodromal AD compared to CU (*η_p_^2^* = 0.13, permutation-adjusted *p* <0.001). There was a significant group by sex interaction only on the connectivity strength of thalamus in the feeder connections (*η_p_^2^*=0.010, *p*=0.025). * *Adjusted p* < 0.05 for sex-difference; # *Adjusted p* < 0.05: CU vs. AD dementia ; ‡ *Adjusted p* < 0.05 for CU vs. Prodromal AD; † *Adjusted p* <0.05 for prodromal AD vs. AD dementia.

In the region-specific alterations of feeder connections (Figure 3), there was significant sex-differences in the connectivity strength of the thalamus among the AD dementia group (0.03 ± 0.07 vs. 0.44 ± 0.9, *η_p_^2^* = 0.02, permutation-adjusted *p* = 0.026).

For the group effects, likewise the rich-club connections, both men and women with AD dementia showed decreased connectivity strength of SPC (*η_p_^2^* = 0.07, permutation-adjusted *p* < 0.001 for women; *η_p_^2^* = 0.13, permutation-adjusted *p* < 0.001 for men) and that of precuneus (*η_p_^2^* = 0.07, permutation-adjusted *p* < 0.001 for women; *η_p_^2^* = 0.03, permutation-adjusted *p* = 0.01 for men) in the feeder connections compared to CU and prodromal AD (Figures 2B,C). Among women, the connectivity strength of hippocampus in the feeder connection was decreased in AD dementia (*η_p_^2^* = 0.03, permutation-adjusted *p* < 0.001) compared to CU or prodromal AD. Among men, the connectivity strength of SPC in the feeder connections was increased in the prodromal AD compared to CU (*η_p_^2^* = 0.13, permutation-adjusted *p* < 0.001).

There was a significant group by sex interaction only on the connectivity strength of thalamus in the feeder connections (*η_p_^2^* = 0.010, *p* = 0.025).

#### 3.2.4. Sex differences in the correlation between the cognitive performance and rich-club organization

The region-specific rich-club connections of the putamen were negatively correlated with the CDR-sum of boxes only in women with AD dementia (*r* = −0.21, permutation-adjusted *p* = 0.006) (Supplementary Figure S2). There were no significant correlations between cognitive function and rich-club connections of the putamen in other cognitive group.

**Figure 4:**
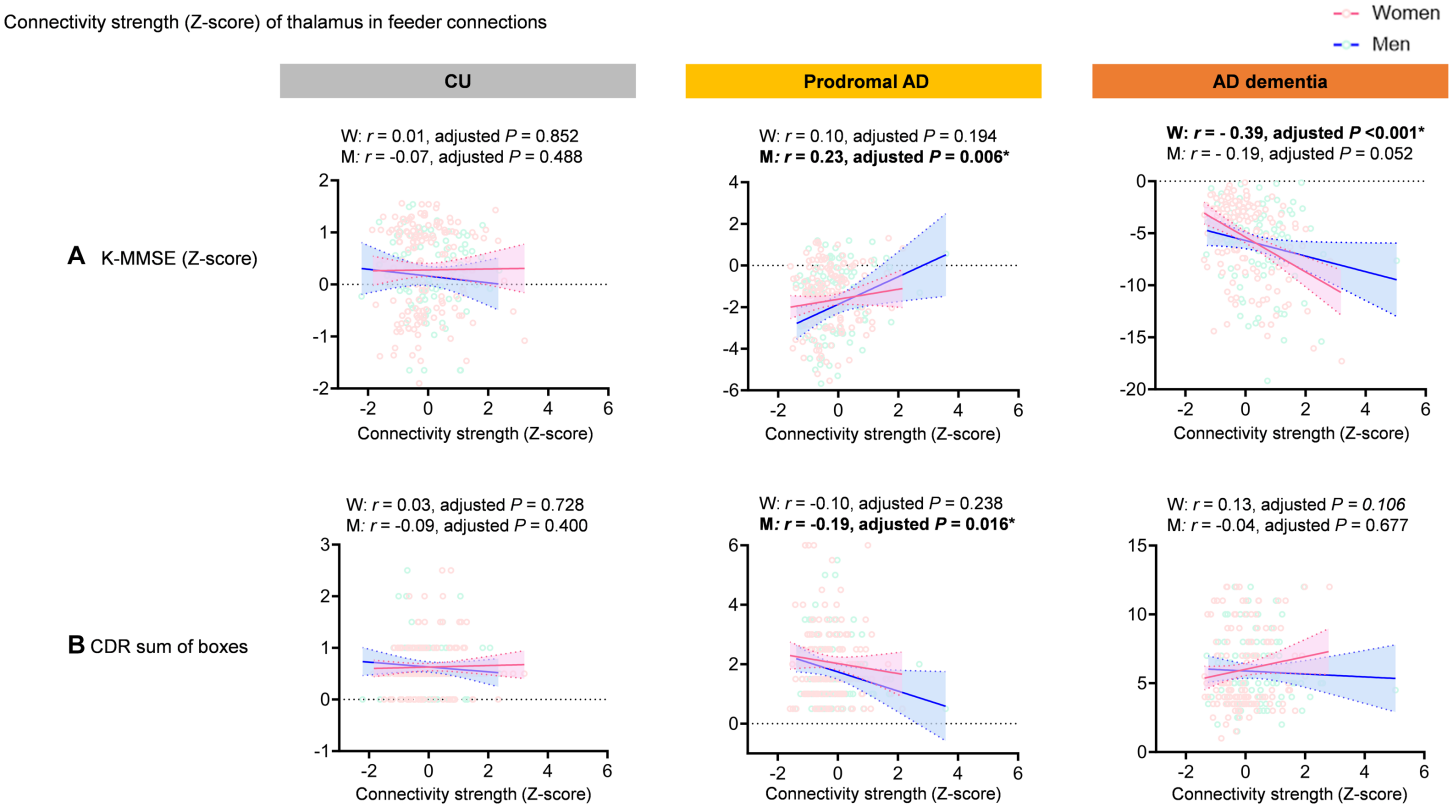
Correlation between feeder connections of the thalamus and cognitive function. The graph shows scatter plot with line of best fit (95% confidence interval). The region-specific feeder connections of the thalamus are positively correlated with the K-MMSE scores (*r* = 0.23, permutation adjusted *p* = 0.006) and negatively correlated with the CDR sum of boxes (*r* = −0.19, permutation adjusted *p* = 0.016) in men with prodromal AD, whereas there is no significant correlation in women with prodromal AD. In the AD dementia, the connectivity strength of the thalamus in the feeder connections are negatively correlated with the K-MMSE scores (*r* = −0.39, adjusted *p* < 0.001) only in women.

However, the region-specific feeder connections of the thalamus are positively correlated with the K-MMSE scores (*r* = 0.23, permutation adjusted *p* = 0.006) and negatively correlated with the CDR sum of boxes (*r* = −0.19, permutation adjusted *p* = 0.016) in men with prodromal AD, whereas there is no significant correlation in women with prodromal AD. In the AD dementia, the connectivity strength of the thalamus in the feeder connections are negatively correlated with the K-MMSE scores (*r* = −0.39, adjusted *p* < 0.001) only in women (Figure 4).

## 4. Discussion

This novel study demonstrated sex differences in the alterations of connectivity strength in the rich-club organization in AD spectrum disease, which was confirmed by amyloid PET.

In the current study, we observed a significant sex difference in the rich-club and feeder connections only in patients with AD dementia. In the AD dementia group, women demonstrated lower connectivity strength of the rich-club and feeder connections than did men. Consistent with our findings, a previous study investigating gender differences in the rich-club organization reported that men demonstrated greater connectivity strength of rich-club nodes demonstrating an advantage in network efficiency in men compared to women (Wang et al., 2019), although this previous study was conducted in young healthy participants.

In terms of group effects of rich-club organizations, we found that both men and women with AD dementia demonstrated decreased structural connectivity of the local connections, compared with patients with CU or prodromal AD. Previous studies have shown that disruptions in the structural connectivity in rich-club organizations may originate from peripheral regions, such as local connections (Daianu et al., 2015; Yan et al., 2018), which are in line with our findings.

Interestingly, compared with CU or prodromal AD, only women with AD dementia demonstrated decreased connectivity strengths of the rich-club and feeder connections, while no differences of rich-club and feeder connections were observed in men with AD dementia. Given that women with prodromal AD and AD dementia demonstrated a similar amount of amyloid deposition with men, our findings may suggest that men may have more preserved rich-club and feeder connections of structural network. This finding may explain greater vulnerability of structural brain network to amyloid deposition in women, which is consistent with previous findings. Earlier research indicates that while the quantity of AD pathology is similar in men and women, the correlation between AD pathology and clinical symptoms in AD dementia is notably stronger in women compared to men (Barnes et al., 2005). In fact, each unit of AD pathology increases the odds of clinical symptoms over 20 times in women, while it only results in a three-fold increase in men. The authors therefore suggested that AD pathology is more likely to be clinically expressed as dementia in women than in men. In addition, previous studies have shown that women with AD have 1 ~ 1.5% higher rate of brain atrophy compared to men (Hua et al., 2010), and women demonstrated increased rate of tau accumulation than men (Smith et al., 2020). The exact causes for the heightened vulnerability of the rich-club organization in women with AD dementia remain uncertain. However, potential factors like sex differences in tau accumulation, comorbidities, cognitive reserve, neurotrophic factors, inflammation, and synapse biology have been proposed as potential contributors to these differences (Ferretti et al., 2018; Laws et al., 2018; Toro et al., 2019).

Significantly, we noticed a group-specific effect related to sex, specifically in the feeder connections, where women exhibited fewer feeder connections compared to men. This finding aligns with a previous study on gender differences in rich-club organization, which reported larger connectivity values, including connectivity strength, in men compared to women for feeder connections. This suggests that disparities in peripheral regions play a pivotal role in understanding gender differences in rich-club organization (Wang et al., 2019).

To uncover potential differences of rich-club organizations between men and women, our study also examined the topological properties of rich-club organization. When we investigated the region-specific alterations in the rich-club connections between men and women, it is noteworthy that men showed significantly higher connectivity strength of the SPC as well as that of the putamen in the rich-club connections than women in the prodromal AD, while the higher connectivity strength of the thalamus in men than women in patients with AD dementia. Consisted with our findings, a previous study showed that the SPC was one of the regions which showed the most significant differences in network topology between women and men based on the 1,053 postmortem brain samples in AD (Sun et al., 2019). In addition, higher rich-club connections of the putamen in men with prodromal AD could be explained by the previous study showing the faster striatal AB accumulation in women than men and more pronounced tau accumulation in women than men (Kim et al., 2022), which may further induce more structural network abnormality in women than men.

It is also noteworthy that both men and women showed higher connectivity strength of the precuneus in the rich-club connections among the prodromal AD group compared to that in CU, but showed lower connectivity strength of the precuneus in AD dementia compared with that in CU or prodromal AD groups. Furthermore, both women and men demonstrate decreased connectivity strength of the SPC in AD dementia group compared to CU or prodromal AD. Alterations of rich-club connections in the precuneus as well as the SPC among prodromal AD or AD dementia could be explained by the facts that the precuneus and the SPC are one of the main regions which are mainly involved in AD (Jacobs et al., 2012; Ota et al., 2022). Consistent with our findings, alterations of structural connectivity in precuneus or SPC among patients with AD were reported in the previous study (Wang et al., 2016; Cao et al., 2020; Prawiroharjo et al., 2020).

Surprisingly, we observed enhanced connectivity strength in the precuneus of patients with prodromal AD. The implications of these structural changes are still a subject of debate. Despite the different methodologies, some researchers propose that increased structural connectivity could indicate a compensatory process in neurodegenerative diseases (Mole et al., 2016; Sheng et al., 2021), consistent with our findings.

Further studies are needed to elucidate to what extent these findings reflect real biological changes of these increased structural connectivity in patients with AD.

It should be noted that we found the significant sex-differences in the connectivity strength of thalamus in the rich-club and feeder connections, indicating that men with AD dementia had greater connectivity strength of thalamus in the rich-club and feeder connections than those in women. No studies have been conducted on the structural connectivity related to the thalamus in AD; however, women with AD dementia have smaller anterior thalamic volumes than do men (Callen et al., 2004), which could be consistent with our findings.

Furthermore, men with AD dementia demonstrated increased region-specific alterations in the feeder connections in the thalamus, compared with men with CU or prodromal AD. Although increased structural connectivity strength could be regarded as a compensatory process as mentioned above, there is limited information about the pathological basis of structural network compensation in AD (Jackson et al., 2019; Sheng et al., 2021); this warrants further research related to the structural connectivity of the thalamus in AD to elucidate sex differences in the thalamus of patients with AD.

Notably, the feeder connections of the thalamus were differently correlated with the cognitive scores in men and women. In men with prodromal AD, the feeder connections of the thalamus were positively correlated with the K-MMSE scores and negatively correlated with the CDR sum of boxes. Considering the thalamus receives and combines neural signals from various parts of the neocortex and is involved in coordinating this information (Aggleton et al., 2016; Alderson et al., 2017), the severity of disruption in the connectivity strength of the thalamus from feeder connections is supposedly correlated with cognitive dysfunction in men with prodromal AD. This finding is supported by previous findings suggestion that cognitive function is associated with connectivity of the thalamus in AD (Aggleton et al., 2016).

However, contrary to our expectations, region-specific feeder connections of the thalamus were negatively correlated with the K-MMSE scores only in women with AD dementia, suggesting that increased thalamic connectivity strength in such patients is associated with poor cognitive function.

The thalamus consists of multiple nuclei that are highly interconnected with the cortical and subcortical areas of the brain (Perry et al., 2021). One possible explanation for this negative correlation between the connectivity strength and cognitive function in women with AD dementia is that increased connectivity strength between certain thalamic nuclei and the cerebral cortex is associated with the inhibitory function of cognition. For example, the suppression of activity in the medial dorsal nucleus of the thalamus may play a vital role in facilitating information processing from the hippocampal formation to the prefrontal cortex, specifically in the memory domain (Yang et al., 2019; Perry et al., 2021). Therefore, the increased connectivity strength of the medial dorsal nucleus in the thalamus is supposedly associated with increased memory impairment in patients with AD dementia.

The current study had some limitations. First, we could not determine the causal relationship between alterations in the brain networks and could not examine cognitive decline because of the cross-sectional nature of the study design. Second, the participants did not have major psychiatric diseases, such as depression or anxiety; therefore, these potential confounding factors were not included in the analysis, which should be considered in future studies. Third, we analyzed the effects of *APOE ε4* on the connectivity strength of the rich-club organizations in patients with AD (Supplementary Figure S3); however, other genetic factors could also affect the structural connectivity of the rich-club organization in AD. Fourth, we performed amyloid PET to confirm amyloid pathology in the brain; nonetheless, we did not consider other pathologies, such as neurofibrillary tangles or Lewy bodies. Finally, although we utilized a permutation-based approach for multiple comparison correction, it is worth noting that this method might be less conservative compared to Bonferroni or False Discovery Rate correction. As a consequence, there is a possibility that it could lead to an increased risk of inflating the Type I error rate.

Nonetheless, the strength of this study is that it investigated the sex differences in the rich-club organizations in patients with AD confirmed by amyloid PET scans, which had not been examined before. Our findings provide important evidence for sex-specific alterations in the structural brain network related to AD, suggesting the necessity of sex-specific strategies in clinical practice.

## Data availability statement

The raw data supporting the conclusions of this article will be made available by the authors, without undue reservation.

## Ethics statement

The studies involving humans were approved by the Institutional Review Board of Samsung Medical Center. The studies were conducted in accordance with the local legislation and institutional requirements. Written informed consent for participation in this study was provided by the participants’ legal guardians/next of kin.

## Author contributions

GK, S-JK, J-KS, and SS contributed to the data conceptualization, analysis, and interpretation. GK and S-JK contributed to the drafting of the manuscript, analysis of the imaging data, and preparation of the figures. YB, YP, HJ, and JK contributed to the data interpretation. All authors have read and approved the final version of the manuscript.

## Funding

This research was supported by a grant of the Korea Health Technology R&D Project through the Korea Health Industry Development Institute (KHIDI), funded by the Ministry of Health & Welfare and Ministry of science and ICT, Republic of Korea (grant number: HU20C0111, HU22C0170, and HU23C1234); the National Research Foundation of Korea (NRF) grant funded by the Korea government (MSIT) (NRF-2019R1A5A2027340); partly supported by Institute of Information & communications Technology Planning & Evaluation (IITP) grant funded by the Korea government (MSIT) (No.2021-0-02068, Artificial Intelligence Innovation Hub); Future Medicine 20*30 Project of the Samsung Medical Center [#SMX1230081]; the “National Institute of Health” research project (2021-ER1006-02) and was supported by the Ewha Womans University Research Grant of 2022.

## Conflict of interest

The authors declare that the research was conducted in the absence of any commercial or financial relationships that could be construed as a potential conflict of interest.

## Publisher’s note

All claims expressed in this article are solely those of the authors and do not necessarily represent those of their affiliated organizations, or those of the publisher, the editors and the reviewers. Any product that may be evaluated in this article, or claim that may be made by its manufacturer, is not guaranteed or endorsed by the publisher.

## Footnotes

^1^ http://www.brain-connectivity-toolbox.net
