# Supplementary material for: Sex differences in the structural rich-club connectivity in patients with Alzheimer’s disease
Source: Front Aging Neurosci. 2023 Sep 13;15:1209027. doi: 10.3389/fnagi.2023.1209027 (PMC10525353; doi:10.3389/fnagi.2023.1209027)
Supplement: Supplementary file 1 [file Data_Sheet_1.pdf]

Supplementary Figure -1

Fig.S1.The normalized weighted rich-club coefficients relative to random networks.

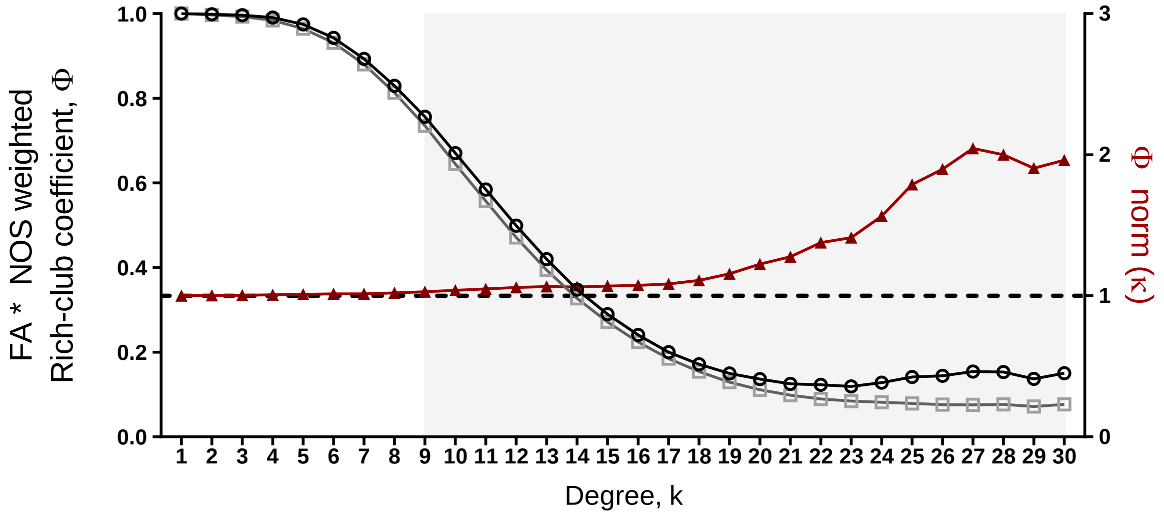

The figure shows the rich-club coefficient values for a range of *k*, for Φ^{w} (Black), $\Phi^{w}{}_{random}$ (light gray) and $\Phi^{w}{}_{norm}$ (red). Φ^{w} is found to be larger than $\Phi^{w}{}_{random}$, which suggests rich-club organization of the structural brain network in this study population (light gray box).

Supplementary Figure -2

Fig.S2. Correlation between rich-cub connections of the putamen and cognitive function

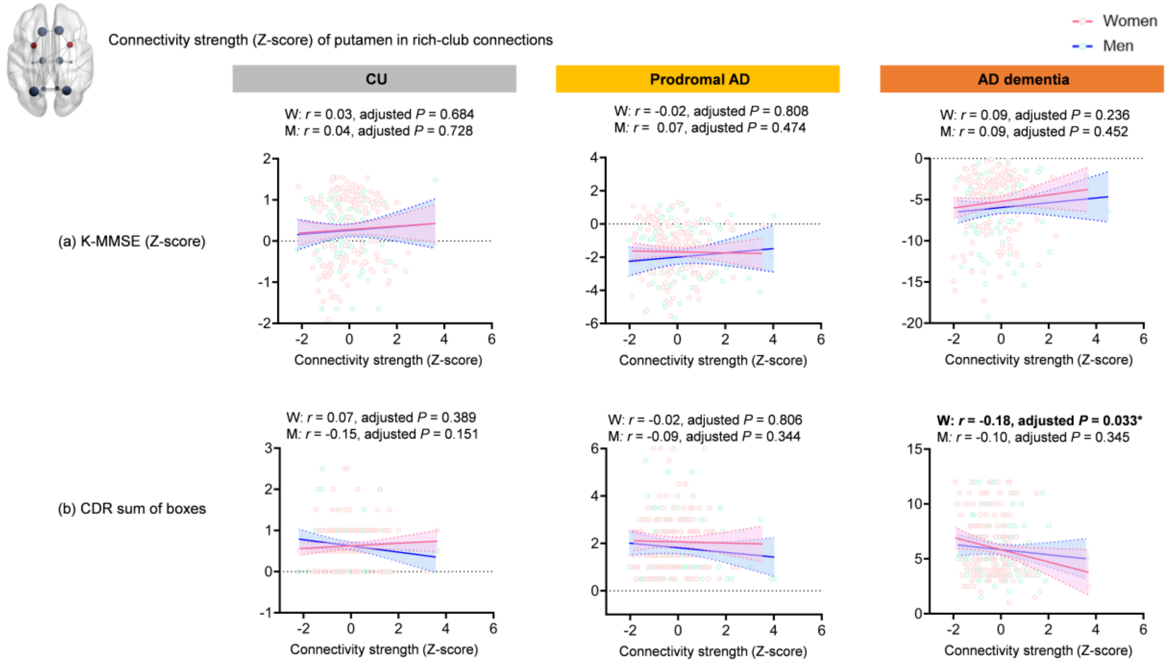

The graph shows scatter plot with line of best fit (95% confidence interval). The region-specific rich-club connections of the putamen are correlated with CDR sum of boxes (*r*=-0.18, permutation adjusted *P*=0.033) only in women with AD dementia.

Supplementary Figure -3

Fig.S3. Sex and *APOE ε4* effects on rich-club connections

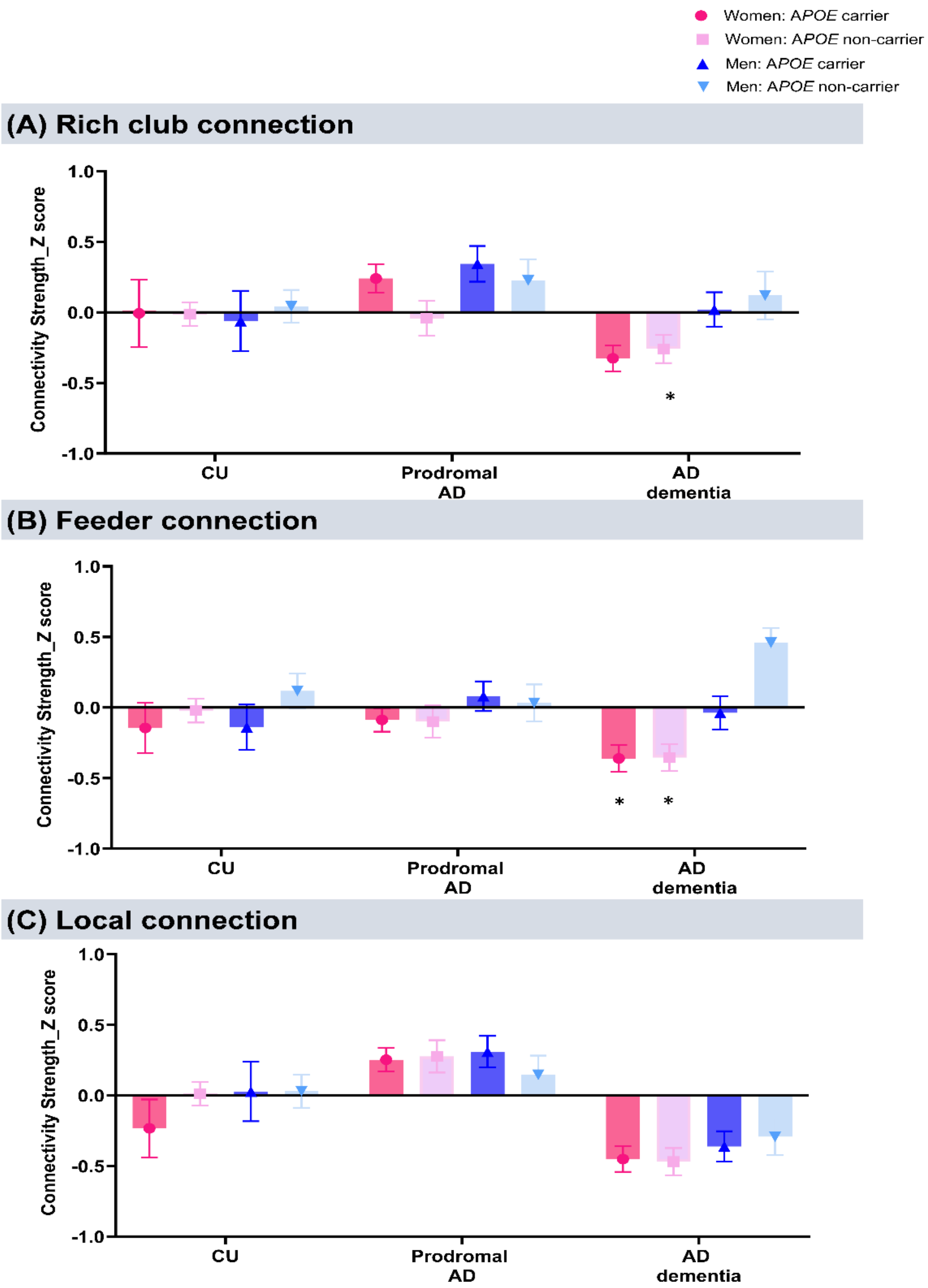

Figure S3 indicates sex and group effects in the region-specific alterations of rich-club connections (mean ± SE).

Especially in patients with AD dementia, women with *APOE ε4* carrier showed lower connectivity strength of rich-club connections compared to men with *APOE ε4* non-carrier,. In addition, women with both *APOE ε4* carrier and non-carrier showed lower connectivity strength of feeder connections compared to men with *APOE ε4* non-carrier in patients with AD dementia.

* $P < 0.05$ if compared to men with *APOE* ***ε4*** non carrier
